# Light drinking versus abstinence in pregnancy – behavioural and cognitive outcomes in 7-year-old children: a longitudinal cohort study

**DOI:** 10.1111/1471-0528.12246

**Published:** 2013-04-17

**Authors:** Y Kelly, M Iacovou, MA Quigley, R Gray, D Wolke, J Kelly, A Sacker

**Affiliations:** a Department of Epidemiology and Public Health, University College LondonLondon, UK; b Institute for Social and Economic Research (ISER), University of EssexColchester, UK; c National Perinatal Epidemiology Unit, University of OxfordHeadington, Oxford, UK; d Department of Psychology and Health Sciences Research Institute, Warwick Medical School, The University of WarwickCoventry, UK

**Keywords:** Alcohol, behaviour, cognitive tests, Millennium Cohort Study, Pregnancy

## Abstract

**Objective:**

To assess whether light drinking in pregnancy is linked to unfavourable developmental outcomes in children.

**Design:**

Prospective population-based cohort.

**Setting:**

UK.

**Population:**

Ten thousand five hundred and thirty-four 7-year-olds.

**Methods:**

Quasi-experimental using propensity score matching (PSM) to compare children born to light (up to 2 units per week) and non-drinkers.

**Main outcome measures:**

Behavioural difficulties rated by parents and teachers; cognitive test scores for reading, maths and spatial skills.

**Results:**

Ordinary least squares (OLS) regression and PSM analyses are presented. For behavioural difficulties, unadjusted estimates for percentage standard deviation (SD) score differences ranged from 2 to 14%. On adjustment for potential confounders, differences were attenuated, with a loss of statistical significance, except for teacher-rated boys' difficulties. For boys, parent-rated behavioural difficulties: unadjusted, −11.5; OLS, −4.3; PSM, −6.8; teacher-rated behavioural difficulties: unadjusted, −13.9; OLS, −9.6; PSM, −10.8. For girls, parent-rated behavioural difficulties: unadjusted, −9.6; OLS, −2.9; PSM, −4.5; teacher-rated behavioural difficulties: unadjusted, −2.4; OLS, 4.9; PSM, 3.9. For cognitive test scores, unadjusted estimates for differences ranged between 12 and 21% of an SD score for reading, maths and spatial skills. After adjustment for potential confounders, estimates were reduced, but remained statistically significantly different for reading and for spatial skills in boys. For boys, reading: unadjusted, 20.9; OLS, 8.3; PSM, 7.3; maths: unadjusted, 14.7; OLS, 5.0; PSM, 6.5; spatial skills: unadjusted, 16.2; OLS, 7.6; PSM, 8.1. For girls, reading: unadjusted, 11.6; OLS, −0.3; PSM, −0.5; maths: unadjusted, 12.9; OLS, 4.3; PSM, 3.9; spatial skills: unadjusted, 16.2; OLS, 7.7; PSM, 6.4.

**Conclusion:**

The findings suggest that light drinking during pregnancy is not linked to developmental problems in mid-childhood. These findings support current UK Department of Health guidelines on drinking during pregnancy.

## Introduction

The link between heavy alcohol consumption during pregnancy and health and developmental problems in children is well established.^1^ The picture for low levels of alcohol consumption is unclear, but an emerging literature suggests that ‘light’ drinking during pregnancy is not linked to detrimental impacts on behavioural or cognitive development during early childhood.^2^–^9^ However, there may be ‘sleeper’ effects, whereby developmental problems associated with mothers' drinking in pregnancy emerge later in childhood.

In the context of women's drinking during pregnancy, randomised controlled trials are not feasible and the evidence base relies on observational studies which typically use regression modelling to account for the effects of confounding factors. The problem with this is that drinking during pregnancy is socially patterned – ‘light’ drinkers are more likely to be socially advantaged, and an advantaged social position is linked to more favourable developmental profiles in young children.^10^ Therefore, we cannot be sure whether the apparent lack of negative impacts of ‘light’ drinking on early child development are ‘real’ or spurious in nature.

Although direct experimental studies cannot be performed here, we can use propensity score matching (PSM) to try to get as close to experimental conditions as possible in an observational study, and thus advance the evidence base. In this article, we use PSM to match children of ‘light’ drinkers and non-drinkers in terms of a range of observed factors, creating matched ‘treatment’ and ‘control’ samples, composed of light drinkers and non-drinkers, respectively. We used data from the Millennium Cohort Study to assess whether light drinking in pregnancy is linked to unfavourable developmental outcomes in 7-year-old children.

## Methods

### Millennium Cohort Study

The Millennium Cohort Study is a nationally representative longitudinal study of infants born in the UK between September 2000 and January 2002. The design and conduct of the survey have been described in detail elsewhere (http://cls.ioe.ac.uk/shared/get-file.ashx?id=598&itemtype=document). The first four sweeps of the survey involved home visits by interviewers when cohort members were aged 9 months, 3, 5 and 7 years. During home visits, interviewers asked questions about the following: drinking in pregnancy, socio-economic circumstances, demographic characteristics, psychosocial environment and cohort members' behaviour. Cognitive assessments were carried out in the home by trained interviewers. Using postal questionnaires, teachers were asked about cohort members' behaviours. Ethical approval for the Millennium Cohort Study was gained from the relevant Ethics Committees and parents gave informed consent prior to the interviews.

### Mothers' drinking

When cohort members were 9 months old, mothers were asked whether they drank alcohol during pregnancy (every day; 5–6, 3–4, 1–2 days per week; 1–2 times per month; less than once per month; never). If the mother drank at least once or twice per week, she was asked: ‘In an average week, how many units of alcohol did you drink?’. If she drank once or twice per month or less than once per month, she was asked: ‘On the days when you did drink alcohol, on average how many units did you drink in a day?’. Mothers were told: ‘By a unit, I mean half a pint of beer, a glass of wine or a single measure of spirit or liqueur’.

During each survey sweep, mothers were asked questions about whether they currently drank alcohol. This information was combined with the information on drinking in pregnancy to identify those mothers who never drank (teetotallers, comprising 12.7% of the sample), those who did not drink in pregnancy, but who did drink alcohol later on in their children's lives (the ‘not in pregnancy’ group, comprising 57.1% of the sample), those who drank no more than 1–2 units per week or per occasion during pregnancy (the ‘light drinking’ group, 23.1% of the sample) and mothers who drank more than this amount (7.2% of the sample). There are no widely agreed definitions for light drinking in pregnancy. Our definition of no more than 1–2 units per week or per occasion is based on the criteria outlined by the National Alcohol Strategy^11^ and is consistent with Department of Health guidelines for drinking during pregnancy.^12^

In this article, we focus on low levels of drinking during pregnancy because, until recently, less attention has been paid to whether or not light drinking is linked to developmental problems in children. We compare children of mothers in the ‘light drinking’ and ‘not in pregnancy’ groups; teetotallers and moderate to heavy drinkers, both of which differ systematically from the other groups in terms of their socio-economic profiles, are omitted from the analysis.

### Behavioural difficulties

Parents and school teachers were asked to complete the Strengths and Difficulties Questionnaire (SDQ), age 4–15 years version (http://www.sdqinfo.com). The SDQ is a validated tool which has been shown to compare favourably with other measures for the identification of difficulties, for example, hyperactivity and attention problems.^13^,^14^ The SDQ covers five domains of social and emotional behaviour, namely conduct problems, hyperactivity, emotional symptoms, peer problems and pro-social behaviour. The sum of scores from the first four domains is used to construct a total difficulties score, where high values indicate ‘behavioural difficulties’.

### Cognitive assessments

Three aspects of cognitive performance were assessed: reading, maths and spatial skills. Reading was tested using the British Ability Scale (BAS) Word Reading assessment, in which the child reads aloud a series of words presented on a card.

Maths skills were assessed using an adapted version of the National Foundation for Educational Research (NFER) Progress in Maths test. Children completed various number-based tasks, covering the topics of number, shape, space and measures, and data handling.

Spatial skills were assessed using the BAS Pattern Construction test, during which the child constructs a design by putting together flat squares or solid cubes with black and yellow patterns on each side. The child's score is based on accuracy and speed.

These assessments use age-related starting points and alternative stopping points to protect the motivation and self-esteem of the child.^15^,^16^

### Potential confounders

Factors that were hypothesised to confound the relationship between mothers' drinking and child behavioural and cognitive development were adjusted for in multivariate models. These factors were: mother's age; whether the pregnancy was planned; whether the mother smoked during pregnancy; whether the cohort member was a first born; ethnicity; lone parent family; and a measure of survey response style^17^ (a composite of non-response to questions including life satisfaction, relationship quality, social networks). Markers of family context collected at sweep 4 (age, 7 years) were: number of children in the household; child's age; highest parental educational qualification; parental income; mother's mental health (K6 Questionnaire);^18^ parental discipline strategies (sum of frequency of ignoring, smacking, shouting, sending to the ‘naughty chair’, removing treats, telling off and bribing); mother's self-rated competence (a better than average parent versus average or below average); mother's self-rated closeness of relationship with child (extremely/very close versus fairly/not very close); whether the mother currently drank alcohol; frequency of someone reading to the child (daily, weekly, less often); whether the child had regular bedtimes.

### Study sample

Behavioural and cognitive outcomes are known to be moderated by multiple births.^19^ A total of 13 363 families participated in sweeps 1 and 4 of the Millennium Cohort Study and, of these, 171 had multiple births. The exclusion of these families reduced the sample to 13 192. Further exclusions were made as a result of: missing data on mothers' drinking in pregnancy (50); mothers who were teetotal during pregnancy (1665); mothers who were moderate to heavy drinkers during pregnancy (943). This gave a sample of 10 534 singleton infants whose mothers were classified as either ‘not in pregnancy’ or ‘light’ drinkers, and who participated in sweeps 1 and 4 of the Millennium Cohort Study. Behavioural difficulties total scores at age 7 years were available from parent ratings (*n* = 10 285) and teacher ratings (*n* = 6816). Cognitive test data were available as follows: reading, *n* = 10 140 cohort members; maths, *n* = 10 280 cohort members; spatial skills, *n* = 10 241 cohort members. Missing data for covariates reduced the samples to: parent-rated behavioural difficulties score, *n* = 9936 (96.6%); teacher-rated behavioural difficulties score, *n* = 6554 (96.2%); reading, *n* = 9689 (95.6%); maths, *n* = 9823 (95.6%); spatial skills, *n* = 9789 (95.6%). Thus, the degree of missing data was relatively small, and did not warrant the implementation of methods such as multiple imputation. Finally, we excluded children with missing test scores in more than one cognitive domain from all analysis of cognitive scores, yielding a final *n* = 9597 for our analysis of cognitive test scores. This procedure meant that coefficients were comparable between all domains of cognitive test performance, and doing this did not affect our results.

Under the PSM procedure, a small number of observations were excluded because some children in the light drinking ‘treatment’ group could not be satisfactorily matched with any child in the not in pregnancy ‘control’ group. Removing these reduced the sample by between 31 and 48 observations. In order for estimates to be fully comparable between OLS and PSM, these small numbers of ‘off-support’ observations were removed from our analysis, and this did not affect the reported estimates.

### Data analysis

We present multivariate estimates using ordinary least squares (OLS) regression, and estimates based on PSM. PSM is used to address the problem that certain types of mothers may be disproportionately selected into the ‘light drinking’ group, and that this selection process may not be dealt with satisfactorily by regression-based models. Under PSM, we calculate the propensity of each mother to be in the ‘light drinking’ group, based on the same covariates as used in the fully adjusted OLS models.

Propensity scores are then used to ‘twin’ each mother in the ‘light drinking’ group with one or more mothers in the ‘not in pregnancy’ group. The two groups (the ‘light drinking’ mothers, on the one hand, and their matched counterparts in the ‘not in pregnancy’ group on the other) have almost identical distributions on all observable characteristics, and differ only in terms of the variable of interest, i.e. light drinking in pregnancy.^20^,^21^ The mean differences in behavioural, reading, maths and spatial skills scores between the two groups may be interpreted as the difference associated with light drinking, net of all the other factors, and net of any selection on observables.

There were gender differences in behavioural difficulties scores and cognitive test scores, and so models are presented for boys and girls separately. Behavioural difficulties and reading, maths and spatial skills scores are standardized to have a mean of zero and a standard deviation (SD) of unity. Model results are presented as a percentage of SD scores for behavioural difficulties and cognitive test scores.

OLS and PSM models are based on cases with complete data on relevant variables using Stata 12 (Stata Corporation, 2011, College Station, TX, USA), with PSM implemented using the psmatch2 and pstest modules, employing Epanechikov kernel matching with bootstrapped standard errors (1000 repetitions).

## Results

### Who participated?

Cohort members whose families participated in Millennium Cohort Study sweep 1, but not in sweep 4, were more likely to be from disadvantaged backgrounds. Their mothers were younger, more likely to be lone parents and had lower incomes compared with mothers who took part in both sweeps (Table S1). Light drinkers were more socio-economically advantaged than mothers in the ‘not in pregnancy’ group (Table S2).

### How do children of light drinkers fare?

**Figure 1:**
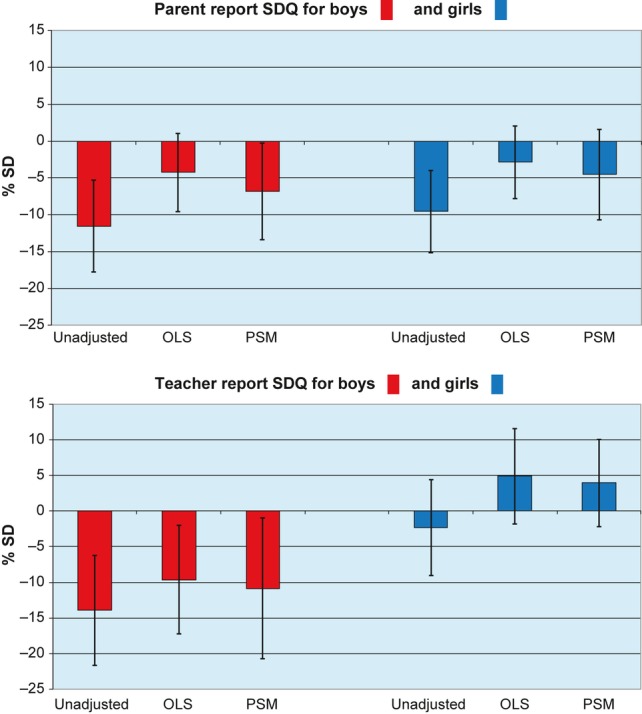
Percentage difference in behavioural difficulties standard deviation (SD) scores: light drinkers versus not in pregnancy. Unadjusted, ordinary least squares (OLS) regression and propensity score matching (PSM) estimates. SDQ, strengths and difficulties questionnaire.

Children born to light drinkers had more favourable (lower) behavioural difficulties scores than those born to mothers in the not in pregnancy group. Unadjusted estimates for percentage SD score differences ranged from 2 to 14%, and were statistically significant, except for teacher ratings of girls' difficulties. Once adjusted for potential confounders, differences were reduced to a similar degree and with a loss of statistical significance in OLS regression and PSM models, except for teacher ratings for boys, which remained statistically significantly different. For boys, parent rated: unadjusted, −11.5; OLS, −4.3; PSM, −6.8; teacher rated: unadjusted, −13.9; OLS, −9.6; PSM, −10.8. For girls, parent rated: unadjusted, −9.6; OLS −2.9; PSM, −4.5; teacher rated: unadjusted, −2.4; OLS, 4.9; PSM, 3.9 (Figure 1).

**Figure 2:**
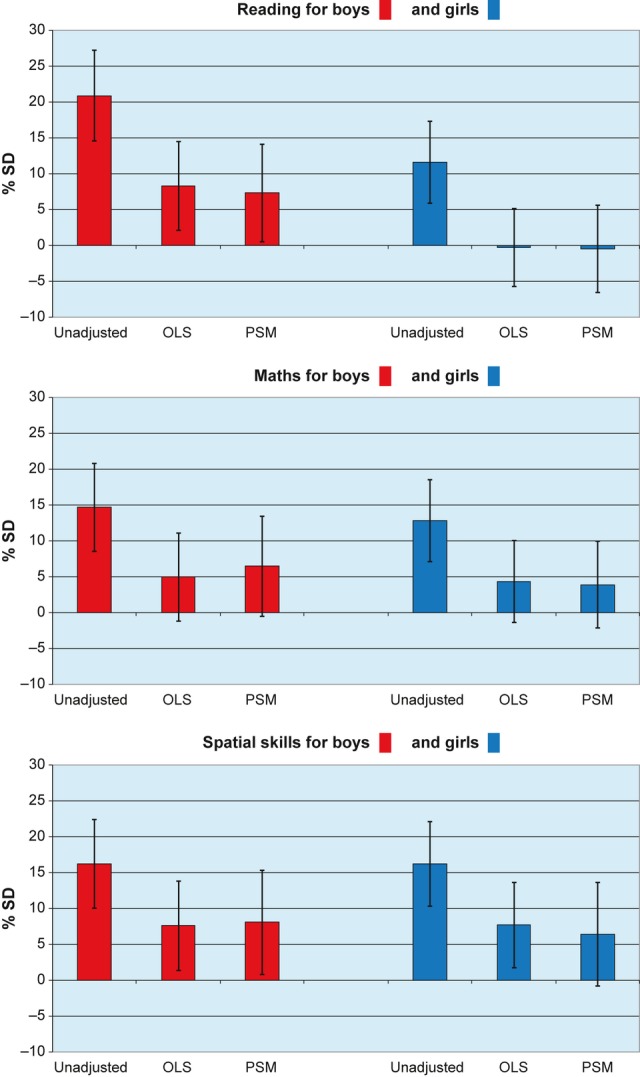
Percentage difference in reading, maths and spatial skills standard deviation (SD) scores: light drinkers versus not in pregnancy. Unadjusted, ordinary least squares (OLS) regression and propensity score matching (PSM) estimates.

Children born to light drinkers were also found to have more favourable (higher) cognitive test scores, between 12 and 21% of an SD score for reading, maths and spatial skills, in unadjusted analysis. After adjustment for potential confounders, estimates were reduced, but remained statistically significantly different from scores of children in the ‘not in pregnancy’ group for reading and for spatial skills in boys. PSM estimates were similar to those from OLS regression models. For boys, reading: unadjusted, 20.9; OLS, 8.3; PSM, 7.3; maths: unadjusted, 14.7; OLS, 5.0; PSM, 6.5; spatial skills: unadjusted, 16.2; OLS, 7.6; PSM, 8.1. For girls, reading: unadjusted, 11.6; OLS, −0.3; PSM, −0.5; maths: unadjusted, 12.9; OLS, 4.3; PSM, 3.9; spatial skills: unadjusted, 16.2; OLS, 7.7; PSM, 6.4 (Figure 2).

The inclusion of birthweight in the models made no substantive difference to the results (data not shown).

## Discussion

### Main findings

In this large, nationally representative study of 7-year-olds, there appeared to be no increased risk of a negative impact of light drinking in pregnancy on behavioural or cognitive development. Prior to statistical adjustment, children born to light drinkers appeared to have more favourable developmental profiles than children whose mothers did not drink during their pregnancies, but, after statistical adjustment, the differences largely disappeared. Our findings from regression models and PSM support the suggestion that low levels of alcohol consumption during pregnancy are not linked to behavioural or cognitive problems during early to mid-childhood.

### Interpretation

Our findings and those of others^4^–^9^ support a null association between light drinking in pregnancy and child development. Furthermore, it does not seem biologically plausible that exposure to small amounts of alcohol *in utero* would have deleterious effects on subsequent development. Children's social and emotional behaviours and cognitive test performances are heavily influenced by the social environment.^10^,^22^,^23^ In this study population, light alcohol consumption during pregnancy is a marker of relative social advantage. Given this, it is perhaps not surprising to find that, in previous work,^2^–^6^ adjustment for socio-economic markers did most to attenuate the observed relationships between light drinking and developmental outcomes. In this article, we used traditional regression modelling alongside a quasi-experimental technique that matched light drinkers and mothers who did not drink during pregnancy on a wide range of socio-economic and psychosocial factors. Our results suggest that measured family socio-economic position and context do not entirely explain the observed relationships between light drinking and children's developmental profiles, and this might be why we see slightly more favourable scores for some markers of child development for children born to light drinkers, but, when apparent, these differences are modest, at around one-tenth of a standard deviation. However, unobserved heterogeneity remains an issue, for example, we were not able to take into account unmeasured influences, such as parental IQ. However, the richness of the data allowed for the control of observed heterogeneity, in particular in aspects of the family environment shown to affect developmental outcomes. Alternative approaches to the assessment of causality in observational studies include the use of instrumental variables, but progress here has been slow, as the identification of plausible instruments for light drinking is difficult. However, a recent study by Lewis et al.^24^ attempted to deal with confounding using a Mendelian randomisation approach, and found effects of fetal genotype on IQ at age 8 years in children of women consuming moderate amounts of alcohol during pregnancy – but not in abstainers or light drinkers.

### Strengths and weaknesses

Data on drinking during pregnancy were collected when cohort members were aged 9 months and, although some studies have reported that the retrospective recall of alcohol consumption is reliable,^25^,^26^ it is possible that the measure used in this study was prone to recall bias. We know that, when not pregnant, about 90% of our sample usually drank alcohol, and around one-third reported drinking during pregnancy, but we did not have data on the timing of drinking during pregnancy. Thus, it is not clear what proportion of women stopped drinking prior to conception or prior to pregnancy recognition, nor is it clear to what portion of their pregnancies mother's reported drinking habits pertain. The light drinking category was heterogeneous in terms of the amounts of alcohol mothers reported consuming, ranging from a very occasional (less than monthly) drink through to the weekly consumption of one or two drinks. We attempted to remove some of the inherent heterogeneity of the non-drinkers by separating out those who did not drink during pregnancy, but who otherwise did drink alcohol, for use as the ‘control’ group. This made the comparison group more similar to the light drinking group in terms of demographic, socio-economic and psychosocial profiles.

Problem behaviours and cognitive deficits in early childhood are strong predictors of later social, behavioural, educational and health outcomes.^27^–^29^ A strength of this study was that we examined data from multi-informants on behavioural development, and on objective measures of cognitive performance for cohort members. These measures have been widely validated and the SDQ has been shown to discriminate clinically diagnosed cases.^30^ However, future work may benefit from the use of more in-depth or wide-ranging assessments of neuropsychological and cognitive function.

## Conclusion

It is accepted that heavy drinking in pregnancy is linked to adverse developmental outcomes in children.^1^ Our findings suggest that drinking not more than one or two units of alcohol per week during pregnancy is not linked to developmental problems in early to mid-childhood, and our findings are consistent with current UK Department of Health guidelines on drinking during pregnancy. However, we remain unclear on what is the level for drinking safely and how this level might be affected by individual susceptibility. Therefore, it may be that the safest option for pregnant women is to avoid drinking during their pregnancies.^12^ Using statistical methods that more effectively deal with confounding in observational studies provides the opportunity to advance knowledge. Nonetheless, further work to tease out whether or not low levels of alcohol consumption during pregnancy are causally linked to developmental problems in childhood is needed.

## Supporting Information

**Table S1.** Socio-economic profiles of mothers lost to follow up from sweep 1 of the Millennium Cohort Study. **Table S2.** Distribution of covariates for ‘light drinkers’ versus ‘not in pregnancy’ groups.
