# Supplementary material for: Light drinking versus abstinence in pregnancy – behavioural and cognitive outcomes in 7-year-old children: a longitudinal cohort study
Source: BJOG. 2013 Apr 17;120(11):1340–7. doi: 10.1111/1471-0528.12246 (PMC4296342; doi:10.1111/1471-0528.12246)
Supplement: Supplementary file 1 [file bjo0120-1340-sd1.pdf]

Table S1. Socioeconomic profiles of mothers lost to follow up from sweep 1 of the MCS

	Productive at sweeps 1 and 4	Lost to follow up at sweep 4
Mother's drinking during pregnancy		
Never	10.0	5.1
Not in pregnancy	55.4	67.2
Light	27.3	19.9
Moderate	5.1	5.2
Heavy/binge	2.3	2.6
Mother's age at the time of birth		
13 to 19	6.1	10.8
20 to 24	14.1	21.9
25 to 29	27.6	27.3
30 to 34	33.1	27.0
35 to 39	16.7	11.1
40 plus	2.4	2.0
Mother smoked during pregnancy		
Yes	19.1	25.8
Lone parenthood		
Yes	11.6	20.3
Family income		
£52,000 or more	7.3	4.9
£32200 - £51999	18.2	10.4
£20800 - £32199	22.3	15.8
£10400 - £20799	28.8	30.4
Less than £10400	16.9	29.1
Don't Know/ Refused	6.5	9.5
Highest parental educational qualification		
Higher degree	7.8	5.6
First degree/diploma	40.0	27.7
A/AS levels	16.3	15.7
GCSE grades A-C	23.7	27.8
GCSE grades D-G	4.7	7.9
Other/overseas	1.3	2.3
None	6.2	13.1

Table S2. Distribution of covariates for light vs. not in pregnancy groups

	Category of drinking	
	Not in Pregnancy n=7061	Light n=2919
Infant's gender		
Male	50.3	51.5
Child's ethnicity		
Minority group	3.3	6.1
*Mother's age at the time of birth******		
13 to 19	8.5	3.9
20 to 24	17.8	8.3
25 to 29	28.4	26.7
30 to 34	29.7	38.1
35 to 39	13.7	20.3
40 plus	1.9	2.8
*Pregnancy planned******		
Yes	56.5	68.4
*Mother smoked during pregnancy******		
Yes	23.1	14.4
Firstborn		
Yes	44.2	43.8
Number of children in the household		
one child	13.8	11.6
two children	48.1	54.0
three plus children	38.1	34.5
*Highest parental educational qualification******		
Higher degree	11.1	19.2
First degree/diploma	36.6	45.6
A/AS levels	18.2	13.4
GCSE grades A-C	23.3	17.4
GCSE grades D-G	4.8	2.1
Other/overseas	1.2	0.5
None	5.1	1.8
*Family income******		
£52,000 or more	9.9	23.1
£32200 - £51999	23.0	30.0
£20800 - £32199	22.7	19.4
£10400 - £20799	24.4	13.8
Less than £10400	11.1	5.4
Don't Know/refused	8.9	8.3

*Mother's K6 score (mean)****	3.0	2.9
*Parental discipline (mean)**	1.5	1.6
Mother's parenting competence		
Better than average	63.1	61.5
Average or below average	36.9	38.5
Mother's closeness to child		
Extremely/ very close	62.6	95.9
Fairly/ not very close	4.2	4.1
*Mother currently drinks****		
Yes	90.3	96.7
*Someone reads to the child**		
Daily	49.6	51.8
Weekly	45.8	44.4
Less often	4.7	3.9
Regular bedtimes		
Never/sometimes	7.3	6.8

$^{*}p<0.05$, $^{**}p<0.01$, $^{***}p<0.001$
